# Serial increase of IL-12 response and human leukocyte antigen-DR expression in severe sepsis survivors

**DOI:** 10.1186/cc10464

**Published:** 2011-09-22

**Authors:** Huang-Pin Wu, Chi-Chung Shih, Chun-Yao Lin, Chung-Ching Hua, Duen-Yau Chuang

**Affiliations:** 1 Division of Pulmonary, Critical Care and Sleep Medicine, Chang Gung Memorial Hospital, 222 Mai-Chin Road, Keelung, 204, Taiwan; 2 Department of Medical Science, Chang Gung University College of Medicine, 259 Wen-Hwa 1st Road, Taoyuan, 333, Taiwan; 3 Department of Emergency, Chang Gung Memorial Hospital, 222 Mai-Chin Road, Keelung, 204, Taiwan; 4 Department of Chemistry, National Chung-Hsing University, 250 Kuo-Kuang Road, Taichung, 402, Taiwan

**Keywords:** interleukin 12, interleukin 6, human leukocyte antigen-DR, peripheral blood mononuclear cells, severe sepsis

## Abstract

**Introduction:**

Sepsis-induced immunosuppression may result in death. The mechanisms of immune suppression include loss of macrophage and monocyte expression of the major histocompatibility complex, increased anti-inflammatory cytokine expression and decreased expression of proinflammatory cytokines. In this study, we sought to determine the mechanisms of immune suppression in severe sepsis by repeated detection.

**Methods:**

We designed this prospective observational study to measure monocyte human leukocyte antigen (HLA)-DR expression, plasma cytokine levels and cytokine responses on days 1 and 7 in stimulated peripheral blood mononuclear cells (PBMCs) of healthy controls and patients with severe sepsis.

**Results:**

Of the 35 enrolled patients, 23 survived for 28 days and 12 died, 6 of whom died within 7 days. Plasma levels of IL-1β, IL-6, IL-10, IL-17, transforming growth factor (TGF)-β1 and TNF-α were higher, but plasma IL-12 level was lower in septic patients than those in controls. Day 1 plasma levels of IL-1β, IL-6, IL-10 and TGF-β1 in nonsurvivors were higher than those in survivors. Day 7 plasma IL-10 levels in nonsurvivors were higher than in survivors. IL-1β response was higher, but IL-12 and TNF-α responses were lower in septic patients than in controls. Day 1 IL-6 response was lower, but day 1 TGF-β1 response was higher in nonsurvivors than in survivors. Plasma IL-6 and IL-10 levels were decreased in survivors after 6 days. IL-6 response was decreased in survivors after 6 days, but IL-12 response was increased. Monocyte percentage was higher, but positive HLA-DR percentage in monocytes and mean fluorescence intensity (MFI) of HLA-DR were lower in septic patients than in controls. MFI of HLA-DR was increased in survivors after 6 days.

**Conclusions:**

Monocyte HLA-DR expression and IL-12 response from PBMCs are restored in patients who survive severe sepsis.

## Introduction

Sepsis is characterized by an acute release of many inflammatory mediators. The balance between pro- and anti-inflammatory mediators influences the survival rate of septic patients. In severe sepsis, immune system failure and sepsis-induced immunosuppression may result in death [1,2]. Loss of macrophage and monocyte expression of the major histocompatibility complex is one of the mechanisms involved, as is diminished surface expression of human leukocyte antigen-DR (HLA-DR) on monocytes [3]. However, not all studies have shown such results [4,5].

A shift from inflammatory to anti-inflammatory cytokines is another mechanism of immune suppression in sepsis. IL-10 level is increased in patients with sepsis and can predict mortality [6]. Nonetheless, IL-10 production from peripheral blood mononuclear cells (PBMCs) in patients with severe sepsis remains unclear. Transforming growth factor (TGF)-β1 can downregulate T-cell, macrophage and granulocyte responses, whereas increased plasma TGF-β1 level is associated with severe disease and mortality in patients with severe pneumonia [7]. Although baseline plasma levels of TGF-β1 are significantly higher in survivors with severe sepsis [8], the correlation of outcome with TGF-β1 production by PBMCs in patients with severe sepsis is lacking.

Diminished proinflammatory cytokine responses also cause immune failure. Low IL-12 production by lipopolysaccharide (LPS)-stimulated PBMCs has been detected in nonsurvivors with severe sepsis [9]. However, plasma IL-12 levels are similar in survivors and nonsurvivors with severe sepsis [10,11]. It may be that high local IL-12 production in infected areas is more important for infection control. The correlation of low IL-12 response with mortality in severe sepsis should be confirmed.

There is a recent emerging cytokine, IL-17, which acts as a potent inflammatory cytokine *in vitro *and *in vivo *[12]. The relationship of circulatory IL-17 level and IL-17 response in humans with severe sepsis is still unknown. IL-1β upregulates adhesion molecule expression and enhances neutrophil and macrophage emigration, while TNF-α enhances proinflammatory cytokine production and natural killer (NK) cell function. The functions of IL-6 in sepsis include induction of acute phage protein production and T- and B-cell differentiation and growth. However, serial responses of IL-1β, TNF-α and IL-6 from PBMCs still need to be elucidated in patients with severe sepsis. Thus, this observational study was designed with repeated blood samplings to determine whether immune suppression is different between survivors and nonsurvivors with severe sepsis.

## Materials and methods

### Participants and definitions

From July 2008 to June 2009, 35 patients who were admitted to a 20-bed ICU in a regional teaching referral hospital for severe sepsis were enrolled in this study. Six nonsurvivors died within 7 days. "Systemic inflammatory response syndrome" (SIRS) was defined if the patient met two or more of the following criteria: (1) body temperature > 38°C or < 36°C, (2) respiratory rate > 20 breaths/minute, (3) heart rate > 90 bpm and (4) white blood cell count > 12,000/μl or < 4,000/μl or > 10% bands. "Sepsis" was defined as SIRS according to a confirmed infectious etiology. To validate experimental findings, 22 men and 8 women with mean age of 60.8 ± 1.9 years old who visited our health evaluation center for examinations were enrolled as healthy controls.

"Severe sepsis" was defined according to the consensus criteria [13,14] for sepsis with dysfunction of one or more organs, such as shock, respiratory failure, acute renal failure, jaundice and thrombocytopenia. "Septic shock" was defined as sepsis-induced hypotension unresponsive to fluid resuscitation. "Respiratory failure" was defined as ventilation dysfunction requiring invasive ventilatory support. "Acute renal failure" was defined as a rapid increase in creatinine level (> 0.5 mg/dl). "Jaundice" was defined as hyperbilirubinemia (total bilirubin > 2 mg/dl). "Thrombocytopenia" was defined as a platelet count < 150,000/μl. Disease severity was assessed according to Acute Physiology and Chronic Health Evaluation (APACHE) II score [15].

Standard treatment according to published guidelines was provided to all patients [16,17]. The Institutional Review Board at Chang Gung Memorial Hospital approved this study (96-1465B), and the patients' close family members provided informed consent. Patients who survived longer than 28 days after ICU admission were defined as survivors. All comorbidities and medical histories were recorded.

### Plasma and peripheral blood mononuclear cell preparation

Whole blood (10 ml) was obtained from each patient at 8:30 AM within 48 hours of admission to the ICU and immediately mixed with heparin. Whole blood from controls was drawn between 8:00 and 8:30 AM and also immediately mixed with heparin. The day of first blood sampling was defined as day 1. A second blood sample was obtained on day 7. Second blood sampling was not done in controls. Plasma samples were obtained from 2 ml of whole blood and stored at -80°C until use. PBMCs were isolated via differential centrifugation over Ficoll-Paque (Amersham Biosciences, Uppsala, Sweden) from 8 ml of residual whole blood within 2 hours of collection.

### Monocyte human leukocyte-DR measurement by flow cytometry

**Figure 1:**
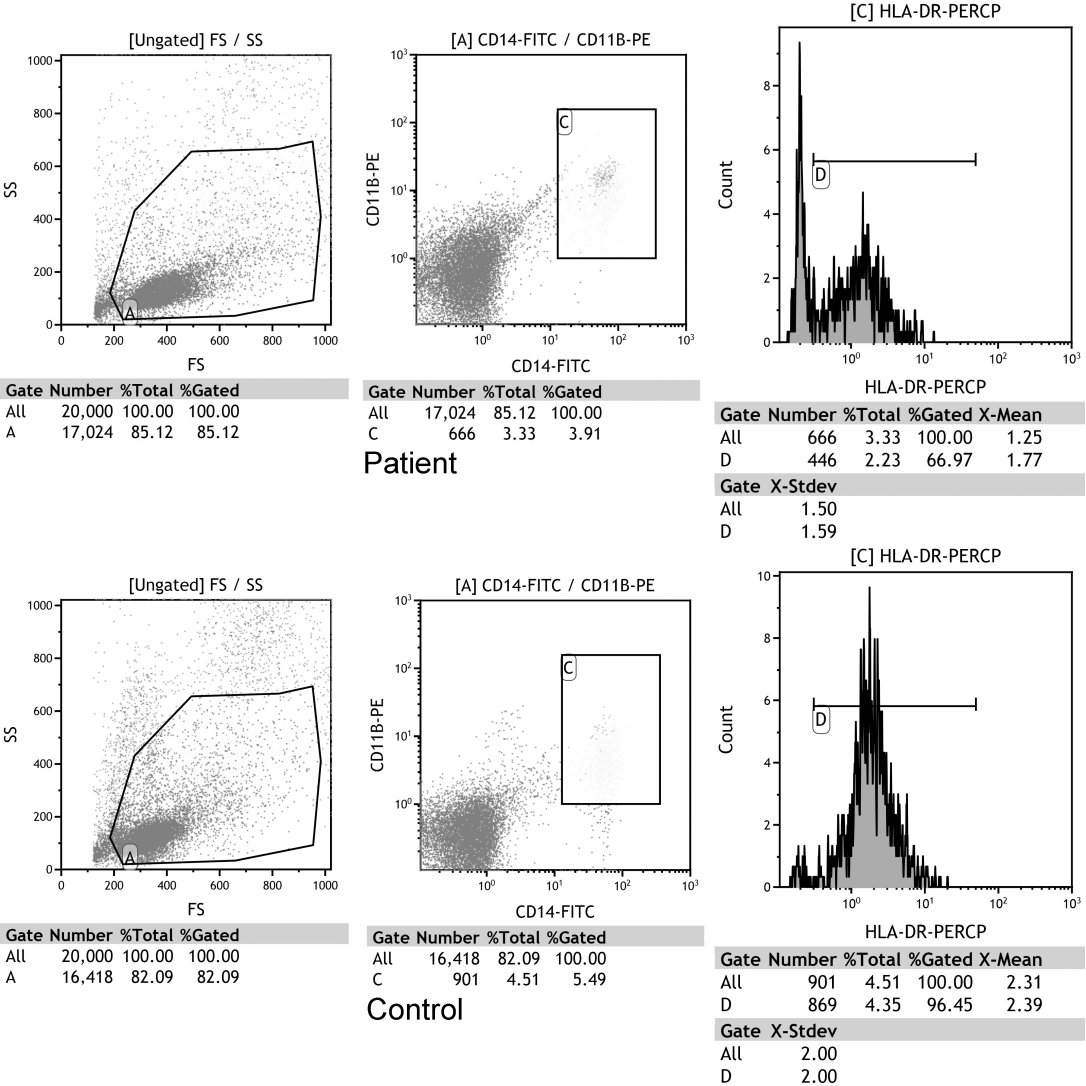
**Flow cytometry of cells from a septic patient and a healthy control**. Monocytes were identified in gated A cells in the scatterplot of forward scatter (FS) and side scatter (SS) with positive CD14 and CD11b (area C). Human leukocyte antigen-DR (HLA-DR) expression is represented in the histogram. Area D was set as positive HLA-DR. In this patient, the percentage of monocytes is 3.33%. HLA-DR was expressed in 66.97% of monocytes. The mean fluorescence intensity of HLA-DR is 1.77. In the control, the percentage of monocytes is 4.51%. HLA-DR is expressed in 96.45% of monocytes. The mean fluorescence intensity of HLA-DR is 2.39. FITC = fluorescein isothiocyanate.

The 2.5 × 10^5 ^PBMCs were suspended in 50 μl of PBS and incubated in the dark for 15 minutes at room temperature with 20 μl of HLA-DR peridinin-chlorophyll-protein complex (HLA-DR_PerCP_), CD11b phycoerythrin (CD11b_PE_) and CD14 fluorescein isothiocyanate (CD14_FITC_) antibodies (BD Biosciences/Pharmingen, San Diego, CA, USA). Then the cells were resuspended in 500 μl of PBS. The monocytes were detected by using a three-color flow cytofluorimeter (Beckman Coulter, CA, USA) with positive CD11b_PE _and CD14_FITC_. Monocyte HLA-DR measurement was expressed as percentages of HLA-DR-positive monocytes and as means of fluorescence intensities (MFIs) related to the entire monocyte population, reflecting the HLA-DR density per cell (Figure 1). Cytofluorimetric analysis was performed with Kaluza Analysis version 1.1 software (Beckman Coulter, Brea, CA, USA).

### Cell culture

The 5 × 10^5 ^PBMCs were plated in two wells of a flat-bottomed 24-well plate (Nunclon™Δ; Nunc A/S, Roskilde, Denmark) in 1 ml of sterile RPMI 1640 tissue culture medium containing 5% heat-inactivated bovine serum, 1 mM L-glutamine (Gibco, Grand Island, NY, USA) and 1 mM sodium pyruvate. The cells in the first well were not stimulated or treated. The cells in the second well were stimulated with 1 pg/μl LPS (Sigma, St Louis, MO, USA). The plate was incubated at 37°C in 5% CO_2 _for 24 hours. Supernatants of the culture wells were sampled and stored at -80°C until use.

### Measurement of cytokine levels

IL-10 levels of plasma and supernatants were measured with a human ELISA kit (Pierce Biotechnology, Rockford, IL, USA), according to the manufacturer's instructions. IL-6, TGF-β1 and IL-17 levels of plasma and supernatants were measured with human ELISA kits (R&D Systems, Inc., Minneapolis, MN, USA), according to manufacturer's instructions. IL-12, TNF- α and IL-1 β levels of plasma and supernatants were measured with human ELISA kits (BD Biosciences), according to the manufacturer's instructions. Cytokine responses were measured as the difference in supernatant levels with and without stimulation. Negative responses were set as 0 pg/ml.

### Statistical analysis

Statistical analysis was performed with Statistical Package for the Social Sciences (SPSS) for Windows version 11.0.1 software (SPSS Inc, Chicago, IL, USA). Differences for continuous variables between two groups were analyzed using the Mann-Whitney *U *test, and differences between categorical variables were analyzed using the χ^2 ^test or Fisher's exact test. Differences for continuous variables in the same subjects were analyzed using the Wilcoxon signed-rank test. *P *< 0.05 was considered statistically significant.

## Results

Of the 35 enrolled subjects with severe sepsis, 23 survived for 28 days and 12 died, 6 of whom died within 7 days. There were no significant differences in age, gender, APACHE II score, medical history, infection source or initial antibiotic susceptibility between survivors and nonsurvivors. The clinical characteristics are shown in Table 1. Nonsurvivors had higher percentages of septic shock, thrombocytopenia and bacteremia than survivors. The percentages of new arrhythmia, gastrointestinal bleeding, acute renal failure and jaundice were similar between the two groups.

**Table 1:** Clinical characteristics in survivors and nonsurvivors

Patient characteristics	Survivors (*n *= 23)	Nonsurvivors (*n *= 12)
Mean age, years (± SEM)	73.0 ± 3.2	73.2 ± 3.3
Males, *n *(%)	15 (65)	9 (75)
Mean APACHE II score (± SEM)	27.1 ± 1.6	32.7 ± 2.1
History, *n *(%)		
Chronic obstructive pulmonary disease	8 (35)	1 (8)
Heart failure	3 (13)	0 (0)
Pneumoconiosis	2 (9)	1 (8)
Bronchiectasis	2 (9)	0 (0)
Hypertension	8 (35)	2 (17)
Diabetes mellitus	4 (17)	3 (25)
Previous cerebral vascular accident	5 (22)	2 (17)
End-stage renal disease	1 (4)	1 (8)
Liver cirrhosis	1 (4)	3 (25)
Active malignancy	3 (13)	5 (42)
Infection source, *n *(%)		
Pneumonia	19 (83)	10 (84)
Urinary tract infection	4 (17)	0 (0)
Catheter infection	0 (0)	1 (8)
Biliary tract infection	0 (0)	1 (8)
Initial antibiotics for pathogens, *n *(%)		
Sensitive	12 (52)	6 (50)
Resistant	6 (26)	6 (50)
No pathogen isolated	5 (22)	0 (0)
Adverse events, *n *(%)		
New arrhythmia	1 (4)	2 (17)
Gastrointestinal bleeding	3 (13)	2 (17)
Acute renal failure	11 (48)	8 (67)
Shock	15 (65)	12 (100)*
Thrombocytopenia	5 (22)	7 (58)*
Jaundice	0 (0)	2 (17)
Bacteremia	1 (4)	4 (33)*

SEM = standard error of the mean; APACHE II = Acute Physiology and Chronic Health Evaluation II. *P < 0.05 compared with survivors by the Mann-Whitney *U *test, χ^2 ^test or Fisher's exact test.

### Plasma cytokine levels and cytokine responses among survivors, nonsurvivors and controls

Day 1 plasma levels of IL-1β, IL-6, IL-10 and TGF-β1 in nonsurvivors were significantly higher than those in survivors (Table 2). There were no differences in day 1 plasma levels of IL-12, IL-17 and TNF-α or in day 7 levels of IL-1β, IL-6, IL-12, IL-17, TGF-β1 and TNF-α between the two groups. Day 7 plasma IL-10 levels were significantly higher in nonsurvivors than in survivors. Day 1 plasma levels of IL-1β, IL-6, IL-10, IL-17, TGF-β1 and TNF-α in septic patients were significantly higher than those in controls. Day 1 plasma IL-12 levels in septic patients were significantly lower than in controls.

**Table 2:** Plasma cytokine levels and cytokine responses on days 1 and 7 in survivors, nonsurvivors and controls

Patient characteristics	Survivors (*n *= 23)	Nonsurvivors (*n *= 12)	All patients (*n *= 35)	Controls (*n *= 30)
Day 1				
Plasma IL-1β	0.1 (0.0 to 2.4)	1.2*(0.0 to 20.6)	0.5 0.0 to 20.6	0.0^† ^(0.0 to 37.7)
IL-1β response	203.8 (0.2 to 220.9)	193.7 (97.4 to 212.8)	203.1 (0.2 to 220.9)	89.3^† ^(0.9 to 209.5)
Plasma IL-6 level	54.6 (4.6 to 442.0)	238.5*(6.5 to 422.0)	73.7 (4.6 to 442.0)	0.8^† ^(0.0 to 64.9)
IL-6 response	451.4 (2.3 to 491.1)	381.4*(88.2 to 444.8)	(389.1 (2.3 to 491.1)	377.8 (2.1 to 670.7)
Plasma IL-10 level	13.9 (0.0 to 110.3)	45.1*(0.0 to 1077.6)	36.5 (0.0 to 1077.6)	3.5^† ^(0.0 to 40.0)
IL-10 response	177.5 (0.0 to 859.7)	283.1 (1.1 to 855.4)	190.0 (0.0 to 859.7)	282.9 (0.0 to 627.8)
Plasma IL-12 level	0.0 (0.0 to 195.9)	6.3 (0.0 to 233.6)	0.0 (0.0 to 233.6)	60.7^† ^(3.4 to 541.6)
IL-12 response	198.4 (0.0 to 1,626.8)	40.7 (0.0 to 1004.9)	152.7 (0.0 to 1,626.8)	(202.0^† ^(0.0 to 1,300.4)
Plasma IL-17 level	8.7 (0.0 to 131.6)	14.4 (0.0 to 197.0)	9.3 (0.0 to 197.0)	0.0^† ^(0.0 to 15.5)
IL-17 response	0.0 (0.0 to 47.3)	0.0 (0.0 to 13.9)	0.0 (0.0 to 47.3)	0.0 (0.0 to 11.4)
Plasma TGF-β1 level	3,498.4 (930.4 to 9,214.1)	5,789.7*(2,501.8 to 15,870.3)	4,151.2 (930.4 to 15,870.3)	3,034.1^† ^(381.5 to 7,890.4)
TGF-β1 response	0.0 (0.0 to 293.9)	96.5*(0.0 to 374.1)	0.0 (0.0 to 374.1)	7.4 (0.0 to 211.2)
Plasma TNF-α level	0.0 (0.0 to 3.5)	0.0 (0.0 to 111.7)	0.0 (0.0 to 111.7)	0.0^† ^(0.0 to 0.5)
TNF-α response	409.2 (30.5 to 515.9)	383.3 (89.3 to 435.4)	399.2 (30.5 to 515.9)	617.3^† ^(0.0 to 725.6)
Day 7	(*n *= 23)	(*n *= 6)	(*n *= 29)	
Plasma IL-1β	0.3 (0.0 to 35.4)	0.6 (0.2 to 2.6)	0.4 (0.0 to 35.4)	
IL-1β response	199.1 (153.7 to 214.9)	201.0 (110.0 to 209.0)	199.6 (110.0 to 214.9)	
Plasma IL-6 level	30.1 (6.0 to 170.2)	68.3 (14.2 to 274.8)	30.1 (6.0 to 274.8)	
IL-6 response	414.0 (32.2 to 470.1)	378.8 (286.5 to 446.3)	401.1 (32.2 to 470.1)	
Plasma IL-10 level	0.0 (0.0 to 89.1)	63.2*(35.4 to 96.9)	5.5 (0.0 to 96.9)	
IL-10 response	181.6 (86.0 to 710.5)	303.2 (0.0 to 480.9)	213.1 (0.0 to 710.5)	
Plasma IL-12 level	0.0 (0.0 to 217.5)	0.0 (0.0 to 43.3)	0.0 (0.0 to 217.5)	
IL-12 response	332.1 (30.5 to 3,097.3)	127.2 (22.7 to 705.8)	329.1 (22.7 to 3,097.3)	
Plasma IL-17 level	6.4 (0.0 to 23.2)	10.6 (0.0 to 70.6)	6.4 (0.0 to 70.6)	
IL-17 response	0.0 (0.0 to 16.2)	0.0 (0.0 to 27.8)	0.0 (0.0 to 27.8)	
Plasma TGF- β1 level	3,164.2 (15,66.7 to 11,100.5)	5,065.2 (2,589.8 to 12,343.1)	3,995.4 (1,566.7 to 12,343.1)	
TGF-β1 response	0.0 (0.0 to 173.7)	0.0 (0.0 to 133.6)	0.0 (0.0 to 173.7)	
Plasma TNF-α level	0.0 (0.0 to 2.8)	0.0 (0.0 to 5.2)	0.0 (0.0 to 5.2)	
TNF-α response	405.4 (198.2 to 521.9)	410.7 (56.4 to 432.7)	406.4 (56.4 to 521.9)	

TGF = transforming growth factor. All data are medians (ranges) in pictograms per milliliter. *P < 0.05 compared with survivors by Mann-Whitney *U *test. ^†^*P *< 0.05 compared with all patients with severe sepsis by Mann-Whitney *U *test.

Day 1 IL-6 response was significantly lower and day 1 TGF-β1 response was significantly higher in nonsurvivors than in survivors. There were no differences in day 1 cytokine responses of IL-1β, IL-10, IL-12, IL-17 and TNF-α or in all detected cytokine responses on day 7 between survivors and nonsurvivors. Day 1 IL-1β responses in septic patients were significantly higher than those in controls. Day 1 IL-12 and TNF-α responses in septic patients were significantly lower than those in controls.

### Plasma cytokine levels and cytokine responses between day 1 and day 7

**Figure 2:**
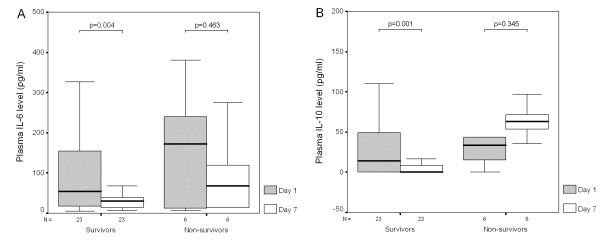
**Plasma IL-6 and IL-10 levels were significantly decreased after 6 days in survivors with severe sepsis**. There was no difference in plasma levels of **(A) **IL-6 and **(B) **IL-10 between day 1 and day 7 in nonsurvivors with severe sepsis.

**Figure 3:**
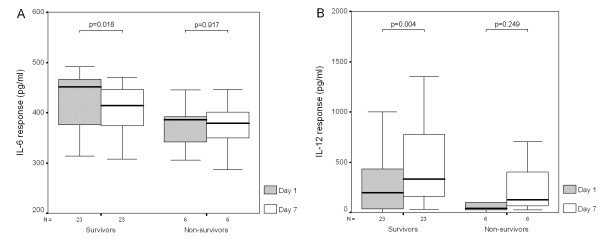
**IL-6 response was decreased while IL-12 response was significantly increased after 6 days in survivors with severe sepsis**. There was no difference in **(A) **IL-6 or **(B) **IL-12 responses between day 1 and day 7 in nonsurvivors with severe sepsis.

Plasma IL-6 and IL-10 levels were significantly decreased in survivors after 6 days (Figures 2A and 2B). There were no changes in plasma levels of IL-1β, IL-12, IL-17, TGF-β1 and TNF-α in survivors. After 6 days, IL-6 responses were also significantly decreased in survivors (Figure 3A), while IL-12 responses were significantly increased in survivors (Figure 3B). There were no changes in cytokine responses of IL-1β, IL-10, IL-17, TGF-β1 and TNF-α in survivors and no changes in all detected plasma cytokine levels and cytokine responses in nonsurvivors after 6 days.

### HLA-DR expression among survivors, nonsurvivors and controls on days 1 and 7

One severe sepsis nonsurvivor did not undergo flow cytometry analysis, owing to insufficient PBMCs. There were no differences in monocyte percentage, positive HLA-DR percentage in monocytes or MFI of HLA-DR between survivors and nonsurvivors on day 1 or day 7 (Table 3). The MFI of HLA-DR was significantly increased in survivors after 6 days. The monocyte percentage and positive HLA-DR percentage in monocytes were similar in survivors between days 1 and 7. However, there were no differences in monocyte percentage, positive HLA-DR percentage in monocytes or MFI of HLA-DR in nonsurvivors between days 1 and 7. Day 1 monocyte percentage in septic patients was significantly higher than that in controls. Day 1 positive HLA-DR percentage in monocytes and MFI of HLA-DR in septic patients were significantly lower than those in controls.

**Table 3:** Monocyte human leukocyte antigen-DR expression in peripheral blood mononuclear cells of survivors, nonsurvivors and controls on days 1 and 7

Patient characteristics	Survivors (*n *= 23)	Nonsurvivors (*n *= 11)	All patients (*n *= 34)	Controls (*n *= 30)
Day 1				
Monocytes (%)	11.3 (0.3 to 39.8)	9.2 (2.7 to 40.5)	11.1 (0.3 to 40.5)	3.6^† ^(1.3 to 9.5)
HLA-DR measurements of positive monocytes (%)	37.8 (1.7 to 66.8)	41.9 (22.3 to 80.7)	41.0 (1.7 to 80.7)	91.7^† ^(57.3 to 98.4)
HLA-DR measurements (MFI)	0.9 (0.6 to 2.9)	0.8 (0.7 to 1.7)	0.9 (0.6 to 2.9)	1.8^† ^(1.4 to 2.6)
Day 7	(*n *= 23)	(*n *= 6)	(*n *= 29)	
Monocytes (%)	9.2 (1.1 to 31.7)	15.6 (3.2 to 25.7)	10.0 (1.1 to 31.7)	
HLA-DR measurement of positive monocytes (%)	45.8 (11.4 to 74.9)	33.4 (18.9 to 71.9)	40.6 (11.4 to 74.9)	
HLA-DR measurements (MFI)	1.2*(0.7 to 1.7)	0.9 (0.7 to 1.2)	1.1 (0.7 to 1.7)	

HLA-DR = human leukocyte antigen-DR; PBMC = peripheral blood mononuclear cell; MFI = means of fluorescence intensity. Data are medians (ranges). *P = 0.004 compared with survivors on day 1 by Wilcoxon signed-rank test. ^†^*P *< 0.05 compared with patients with severe sepsis by Mann-Whitney *U *test.

## Discussion

Inflammation is an important response to infection, as it induces clearing of invasive pathogens. Monocytes link innate immunity and are key immune cells in sepsis. As antigen-presenting cells, monocytes and macrophages are the first host defense against infection. Reduced HLA-DR expression reflects sepsis-induced immunosuppression and is associated with clinical outcome [8,18]. Lymphocyte apoptosis is increased in CD4+ and CD8+ T cells in septic compared to nonseptic patients [19]. Decreased lymphocyte counts result in an increased percentage of monocytes in PBMCs. Decreased HLA-DR expression in septic patients is well-known [3]. All of the above data are similar to our findings of higher monocyte percentage and lower HLA-DR expression in patients with severe sepsis compared to healthy controls.

Our current study demonstrates that patients with serial increases of HLA-DR expression in monocytes have favorable outcomes in severe sepsis. A weak trend of HLA-DR expression recovery is associated with increased risk of secondary infection in ICU patients, such that the lower the slope of HLA-DR expression recovery is, the higher the incidence of first secondary infection will be [20]. Thus, recovery of HLA-DR expression may represent recovery from sepsis-induced immunosuppression and improved outcome. In this study, there was no difference in monocyte percentage, positive HLA-DR percentage in monocytes and MFI of HLA-DR between survivors and nonsurvivors on day 1 or day 7. This is different from the results of the studies by Hynninen *et al*. [21] and Lekkou *et al*. [8], who reported that HLA-DR expression upon admission is significantly higher in survivors with severe sepsis than in nonsurvivors. However, our results are similar to those reported by Monneret *et al*. [22] in that HLA-DR expression was not significantly different on days 1 and 2 between survivors and nonsurvivors with septic shock. The conflicting results may be due to different times of blood sampling (early vs late). Among the study patients, the time of blood sampling was upon ICU admission, not in the early stage of severe sepsis. In this investigation, some patients were admitted to the ICU while the sepsis was in the late stage and some patients were admitted to the ICU when the sepsis was in the early stage. The analysis of trends of HLA-DR expression in each subgroup may be better than the analysis of differences between subgroups.

Weighardt *et al*. [23] found impaired preoperative monocyte IL-12 secretion in patients who developed fatal postoperative sepsis. Weighardt *et al*.'s data suggest that partial preoperative monocyte paralysis may impair host defenses against postoperative infection, resulting in increased risk of lethal sepsis. Our current study is the first to report serial increases in IL-12 response from PBMCs in survivors with severe sepsis. This result is similar to the results reported by Stanilova *et al*. [9], who showed that survivors with severe sepsis produce more IL-12 from LPS-stimulated PBMCs than nonsurvivors. The main immunological function of IL-12 is to enhance native T-lymphocyte differentiation to type 1 T helper (Th1) cells. Th1 cells secrete interferon-γ, which regulates macrophage and NK cell activation, stimulates immunoglobulin secretion by B cells and enhances Th1 cell differentiation. Thus, increased IL-12 response in patients with severe sepsis may exert a protective effect by increased cellular immunity and phagocytic functions.

IL-10 can inhibit monocyte and macrophage proinflammatory cytokine production, Th1 cell differentiation and NK cell function. A sustained high plasma IL-10 level is the main predictor of severity and poor outcome in patients with severe sepsis [11,18]. Our current study shows the same results. Plasma IL-10 levels in nonsurvivors with severe sepsis were higher than those in survivors on days 1 and 7. Moreover, plasma IL-10 levels in survivors significantly decreased from days 1 to 7, whereas plasma IL-10 levels in nonsurvivors did not change. There are reports of similar results. Ozbalkan *et al*. [24] detected a second peak serum IL-10 level in nonsurvivors with burn-induced sepsis. Stanilova *et al*. [9] reported low IL-10 production after LPS stimulation in survivors with severe sepsis. However, in our current study, we did not find similar results. More studies are needed to elucidate the relationship of IL-10 response with outcome in patients with severe sepsis.

Plasma IL-6 level is also a marker for predicting infection and survival in patients with sepsis [11,25]. Above results support the concept that IL-6 level in the early stage of severe sepsis can be a good predictor of mortality. In our current study, plasma IL-6 levels on day 1 in nonsurvivors were higher than those in survivors. Moreover, plasma IL-6 levels in survivors were significantly decreased from day 1 to day 7. The decreasing trend in plasma IL-6 levels was not found in nonsurvivors with severe sepsis. IL-6 responses from PBMCs of survivors significantly decreased from day 1 to day 7. This indicates that IL-6 may be a marker, not an actual mediator, of defense against pathogens.

Plasma TGF-β1 levels upon admission are significantly higher in nonsurvivors of severe sepsis than in survivors [11]. Moreover, TGF-β1 responses from PBMCs were higher in nonsurvivors than in survivors. The anti-inflammatory effects of TGF-β1 may act as a mediator of sepsis-induced immunosuppression. In the current study, there were no differences in plasma IL-17 levels and IL-17 responses between survivors and non-survivors on day 1 or day 7. These results are similar to those reported by White *et al*. [26]. In our present study, plasma IL-17 levels in septic patients were higher than those in controls, but IL-17 responses between patients and controls were similar. IL-17 plays an important role in infection and inflammation [27]. Thus, septic patients need more IL-17 to help eradicate pathogens, and the ability to produce IL-17 did not diminish. More investigations are needed to determine the role of IL-17 in severe sepsis.

In this work, the capacity to produce IL-12 and TNF-α from PBMCs of patients with severe sepsis was diminished, whereas the release of IL-10 was not affected. These findings are well-known [3]. Plasma IL-6, IL-10 and TGF-β1 levels were higher, and plasma IL-12 levels were lower, in septic patients than in controls. These findings are similar to those of a previous study [11]. There is overwhelming support for increased IL-1β expression in severe sepsis. However, anti-IL-1β treatment does not increase survival in patients with severe sepsis [28,29]. In this work, there was no change in plasma IL-1β level and IL-1β response between day 1 and day 7 in survivors or nonsurvivors. This suggests that IL-1β is important for inflammation but is not associated with mortality.

There is one limitation in this study. Fewer than 10 nonsurvivors were alive for more than 7 days. This result is indicative of a relatively low power of statistical analysis in comparisons between days 1 and 7 in the nonsurvivor group and between groups of survivors and nonsurvivors on day 7.

## Conclusions

In our present study, we found that monocyte HLA-DR expression and IL-12 response from PBMCs recovered in survivors of severe sepsis. The role of IL-6 in sepsis may be as a parameter of disease severity. Persistently high plasma IL-10 level may cause sepsis-induced immunosuppression and death. These findings aid in the understanding of immune system reactions during the process of severe sepsis.

## Key message

• Monocyte HLA-DR expression and IL-12 response from PBMCs are recovered in survivors with severe sepsis.

## Abbreviations

APACHE II: Acute Physiology and Chronic Health Evaluation II; BSA: bovine serum albumin; ELISA: enzyme-linked immunosorbent assay; HLA: human leukocyte antigen; IL: interleukin; LPS: lipopolysaccharide; MFI: means of fluorescence intensity; NK: natural killer cell; PBMC: peripheral blood mononuclear cell; PBS: phosphate-buffered saline; SIRS: systemic inflammatory response syndrome; SPSS: Statistical Package for the Social Sciences; Th: T helper cell; TGF: transforming growth factor; TNF: tumor necrosis factor.

## Competing interests

The authors declare that they have no competing interests.

## Authors' contributions

HPW designed the study and wrote the manuscript. CCS helped to collect blood samples and clinical data. CCS, CYL, CCH and DYC participated in data analysis and interpretation of the results.
